# Volumetric versus Element-scaling Mass Estimation and Its Application to Permo-Triassic Tetrapods

**DOI:** 10.1093/iob/obae034

**Published:** 2024-09-13

**Authors:** M A Wright, T J Cavanaugh, S E Pierce

**Affiliations:** Museum of Comparative Zoology and Department of Organismic and Evolutionary Biology, Harvard University, Cambridge, Massachusetts 02138, USA; Museum of Comparative Zoology and Department of Organismic and Evolutionary Biology, Harvard University, Cambridge, Massachusetts 02138, USA; Harvard Extension School, Harvard University, Cambridge, Massachusetts 02138, USA; Museum of Comparative Zoology and Department of Organismic and Evolutionary Biology, Harvard University, Cambridge, Massachusetts 02138, USA

## Abstract

Size has an impact on various aspects of an animal's biology, including physiology, biomechanics, and ecology. Accurately and precisely estimating size, in particular body mass, is therefore a core objective of paleobiologists. Two approaches for estimating body mass are common: whole-body volumetric models and individual element-scaling (e.g., bones, teeth). The latter has been argued to be more accurate, while the former more precise. Here, we use minimum convex hulls (MCHs) to generate a predictive volumetric model for estimating body mass across a broad taxonomic and size range (127 g – 2735 kg). We compare our MCH model to stylopodial-scaling, incorporating data from the literature, and find that MCH body mass estimation is both more accurate and more precise than stylopodial estimation. An explanation for the difference between methods is that reptile and mammal stylopod circumference and length dimensions scale differentially (slope 1.179 ± 0.102 vs. 1.038 ± 0.031, respectively), such that reptiles have more robust bones for a given size. Consequently, a mammalian-weighted stylopodial-scaling sample overestimates the body mass of larger reptiles, and this error increases with size. We apply both estimation equations to a sample of 12 Permo-Triassic tetrapods and find that stylopodial-scaling consistently estimates a higher body mass than MCH estimation, due to even more robust bones in extinct species (slope = 1.319 ± 0.213). Finally, we take advantage of our MCH models to explore constraints regarding the position of the center of mass (CoM) and find that relative body proportions (i.e., skull:tail ratio) influence CoM position differently in mammals, crocodylians, and Permo-Triassic tetrapods. Further, we find that clade-specific body segment expansion factors do not affect group comparisons but may be important for individual specimens with rather disproportionate bodies (e.g., the small-headed and large-tailed *Edaphosaurus*). Our findings suggest that the whole-body volumetric approach is better suited for estimating body mass than element-scaling when anatomies are beyond the scope of the sample used to generate the scaling equations and provides added benefits such as the ability to measure inertial properties.

## Introduction

The size of an organism influences many aspects of its biology, including physiology (e.g., metabolic rate) (West et al. 1997; White et al. 2022), biomechanics (e.g., posture, gait) (Alexander et al. 1979; Alexander and Jayes 1983; Heglund and Taylor 1988; Biewener 1989), and ecology (e.g., species diversity, population density) (Damuth 1981; McClain and Boyer 2009). For over a century (Gregory 1905), paleobiologists have sought to estimate the size of extinct animals in order to uncover a more comprehensive understanding of the geologic past. Trends in body size can help elucidate the origin of novel traits, such as endothermy in mammals (McNab 1978) or the mammalian jaw and middle ear bones (Lautenschlager et al. 2018), as well as the drivers of macroevolutionary patterns, such as the competitive replacement of small-bodied pterosaurs by birds during the Early Cretaceous (Benson et al. 2014). In particular, estimating the body mass of an animal allows researchers to quantify inertial properties, or the resistance to motion during movement, of individual musculoskeletal structures and the body as a whole (Bishop et al. 2020; Lautenschlager 2020). Inertial estimates are key for multibody dynamic simulations to approximate functional traits of living and fossil taxa, such as feeding and locomotor behaviors (e.g., Anderson and Westneat 2007; Snively et al. 2013; Bishop et al. 2021). These methods are powerful in their capacity to shed light on the biology of animals that are hard to measure experimentally or have been extinct for hundreds of millions of years.

Two methodological approaches are commonly used to estimate body mass from skeletal material: the scaling of individually preserved elements, such as bones and teeth (e.g., Damuth and MacFadden 1990; Janis 1990; Campione and Evans 2012; Huang et al. 2024), and volumetric-based reconstructions based on whole skeletons (e.g., Coatham et al. 2021b; Sellers et al. 2012; Bates et al. 2015; Nyakatura et al. 2015; Otero et al. 2019; Macaulay et al. 2023). Element-scaling focuses on the dimensions of individual hard tissue structures (e.g., lengths, widths), while volumetric-based reconstructions instead consider the maximal amount of hard tissue information available for a given specimen or species. Element-scaling thus typically includes much larger sample sizes. Volumetric-based methods range from constructing clay models to measure displacement in water to more recent computational advances that simulate soft tissues around skeletons to generate volumetric measurements and subsequently body mass (see Brassey 2016 and Campione and Evans 2020 for a detailed history of the specific implementations of these two approaches).

Recently, Campione and Evans (2020) discussed the difference between accuracy (whether an estimate measures the true desired value) and precision (the amount of error around an estimated value) in the context of element-scaling and volumetric-based body mass estimation. They argue that volumetric-based methods do not test accuracy but instead test the ability of the investigator to reconstruct a body and subsequently estimate its mass from skeletal material. However, Sellers et al. (2012) demonstrated that volumetric-based estimation can be similarly objective using predictive regression with minimum convex hulls (MCHs) of fully articulated skeletons and therefore may serve as a test of accuracy (see also Coatham et al. 2021b and Macaulay et al. 2023). Further, a key aspect of MCH volumetric-based reconstruction is methodological validation by applying the same reconstruction steps to extant taxa with known body mass (Hutchinson 2012). Campione and Evans (2020) additionally suggest that volumetric-based methods should generate more precise estimates than element-scaling, which has yet to be tested.

Here, we compare the accuracy and precision of these two body mass estimation methods among a sample of extant quadrupedal tetrapods where body mass is either known or reasonably well approximated. We extend and standardize existing datasets for stylopodial-scaling (Campione and Evans 2012) and MCH estimation (Sellers et al. 2012; Coatham et al. 2021b) by creating volumetric models of crocodylians with known body masses and additionally measure limb dimensions. We then determine accuracy and precision for each stylopodial-scaling (*n* = 266) and MCH estimation (*n* specimens = 33, *n* species = 25) by calculating % prediction error (PPE) between specimen body mass and predicted body mass. We further explore how stylopod “robustness” scales within and across clades as a potential explanation for differences in accuracy and precision between estimation methods. Using linear regression equations derived from each estimation method, we apply our findings to the fossil record to reconstruct the body mass of 12 extinct Permo-Triassic tetrapods. Finally, we take advantage of our volumetric models to briefly explore whether there is a correlation between gross body proportions and center of mass (CoM) position across both extant and extinct quadrupedal tetrapods.

## Methods

### Study sample

Our extant dataset included 33 quadrupedal tetrapods that ranged in body mass from 127 g to 2734.9 kg (Supp. File 2). CT scans of 12 crocodylians (7 *Crocodylus niloticus*, 3 *Crocodylus moreletii*, 1 *Caiman crocodylus*, and 1 *Osteolaemus tetraspis*) were downloaded from the public repository CrocBase (https://osf.io/x38nh) and segmented into 3D meshes using Mimics (Materialise NV, Leuven, Belgium). CrocBase specimens were chosen based on completeness of material and quality of osteological definition in the scan. Of note, some crocodylian specimens from the same repository were used in a recent study evaluating the evolution of CoM in bird-line archosaurs (Macaulay et al. 2023). Whole-body 3D meshes of seven mammals (*Mus musculus, Hemicentetes semispinosus, Martes melampus, Nyctereutes procyonoides, Procyon lotor, Paguma larvata*, and *Acinonyx jubatus*) were downloaded from the Dryad Digital Repository (Coatham et al. 2021a). Whole-body volume estimates for 14 additional mammals were incorporated from Sellers et al. (2012). Crocodylian body masses ranged from 127 g to 45 kg, and mammal body masses ranged from 21.5 g to 3734.9 kg.

In addition to our extant sample, we used photogrammetry to generate 3D whole-body meshes of the skeletons of six articulated Permo-Triassic tetrapod fossils on display at the Harvard Museum of Natural History (Supp. File 2). These included one stem amphibian (*Eryops megacephalus*), one stem amniote (*Diadectes tenuitectus*), and four stem mammals (*Edaphosaurus boanerges, Ophiacodon uniformis, Dimetrodon milleri*, and *Dinodontosaurus turpior*), each with unique morphologies not represented among extant diversity. Two stem mammals previously µCT scanned for another study (*Procynosuchus delaharpeae* and *Scaloposaurus constrictus*) (Jones et al. 2020), as well as the stem amniote *Orobates pabsti* (Nyakatura et al. 2015), were added to this dataset in addition to information from the literature for the pareiasaur *Bradysaurus baini* (Brandt et al. 2023) and two additional stem mammals *Tapinocaninus pamelae* (Romano and Rubidge 2019) and *Lisowicia bojani* (Romano and Manucci 2019). In total, we included information from 12 extinct Permo-Triassic tetrapods, with an emphasis on stem mammals (or non-mammalian synapsids).

### Extant volumetric-based estimations

**Fig. 1:**
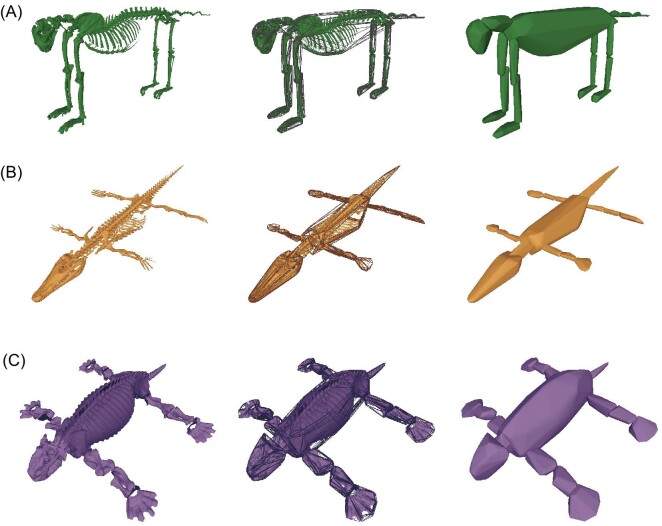
MCH reconstructed models for a representative (A) mammal (*Acinonyx jubatus*; convex hulls from Coatham et al. 2021b), (B) crocodylian (*Crocodylus niloticus*; convex hulls produced for this study), and (C) Permo-Triassic tetrapod (*Dinodontosaurus turpior*; convex hulls produced for this study). From left to right for each model: The articulated skeleton in a standard pose, the skeletal model wrapped in a transparent MCH, and the skeletal model wrapped in an opaque, filled MCH.

Our extant dataset (*n* specimens = 33, *n* species = 25) included directly calculating volumetric estimates of body mass for 19 specimens (12 crocodylians and the 7 mammals from Coatham et al. 2021b) and incorporating the 14 volume estimates reported in Sellers et al. (2012). For the 19 extant specimens where we performed the volumetric estimates directly, 3D skeletal meshes were first standardized into a reference pose using 3-Matic (Materialise NV, Leuven, Belgium) (Fig. 1). For mammals, we used a parasagittal pose with the skull oriented cranially, the tail straightened and oriented caudally, and with the limbs directed straight underneath the body. For crocodylians, the skull was again oriented cranially and the tail straightened and oriented caudally, but the limbs were instead splayed laterally out to the side (a “T”-pose). Osteoderms were not consistently present in the crocodylian scans, so they were removed where visible. For each specimen, the minimum following 16 body segments were exported as separate 3D mesh files: skull, neck, trunk (including the dorsal vertebral column, ribs, pectoral girdles, and pelves), tail, and the stylopodia, zeugopodia, and autopodia for each limb. For the mammals from Coatham et al. (2021b), the original hulls were used, which divided the tail into multiple segments, while we instead straightened the tails of the crocodylians and fossils prior to forming a single tail convex hull (e.g., see Fig. 1). These approaches are both in the original spirit of generating a minimally encompassing convex hull model of the skeleton and are not expected to have noticeable influence on the broader comparisons made in this study.

With the exported mesh files, we created a Python script (https://github.com/awrightmark/MCH_MassCoM) that uses the “trimesh” library (https://trimesh.org/) to generate an MCH around each body segment and compute its volume (Fig. 1). The volume for each segment was added together to produce a whole-body volume for each animal. For the 14 mammals from Sellers et al. (2012), volumes were reported in the original publications and entered into our pipeline at this stage. We multiplied the whole-body volume by a standard density estimate of 1000 kg/m^3^ (equivalent to water). Known whole-body density estimates of extant taxa range from 893 kg/m^3^ in horses (Buchner et al. 1997) to 1080 kg/m^3^ in crocodylians (Cott 1961). However, we found that varying density within this range has negligible impact on the main argument herein (Supp. File 1), which is in accordance with previous work (Macaulay et al. 2017; Durston et al. 2022), so we use 1000 kg/m^3^ to simplify the scope of this study.

An important caveat to note regarding the Sellers et al. (2012) dataset (*n* = 14) is that the body mass values were derived from scaling equations, as this information was not recorded for each specimen in the museum collections database. Body mass for these animals was approximated using species-specific measures such as shoulder height, femur length, or total body length. We regard this species-specific method for approximating body mass as considerably different from a “universal” tetrapod-wide scaling equation that we compare to volumetric-based estimation throughout this study.

We established an MCH scaling equation among extant taxa (*n* = 33) using ordinary least squares (OLS) regression by regressing log10 body mass against log10 MCH-estimated body mass (Warton et al. 2006). We additionally computed an OLS regression following Sellers et al. (2012), where the intercept is forced through (0,0) and the slope is interpreted to be an “expansion factor.” To assess whether a logged scaling equation or an expansion factor better fit our dataset, we generated and examined quantile–quantile plots. Finally, to account for potential phylogenetic relatedness in our dataset, we performed phylogenetic generalized least squares (GLS) regressions, placing our within-species crocodylians as polytomies. Our phylogeny was created by uploading a species list into timetree.org (Kumar et al. 2022). The informativeness of accounting for phylogeny was assessed by comparing phylogenetic GLS regression lines to the 95% confidence bands of non-phylogenetic GLS regressions, in accordance with previous approaches (Campione and Evans 2012; Sellers et al. 2012). OLS regressions were performed in base R (R Core Team 2024), while GLS regressions used the “gls” function from “nlme” (Pinheiro and Bates 2000).

### Extant stylopodial-scaling estimation

We measured humerus and femur minimum midshaft circumference of the 33 extant specimens in the MCH analysis and added these to 233 amniote stylopod dimensions reported in Campione and Evans (2012) for a total extant sample of 266 specimens. We excluded amphibians from the Campione and Evans (2012) dataset due to their small body mass, as well as any duplicate taxa, keeping those we measured directly. Of note, the reptilian sample from Campione and Evans (2012) includes only two specimens with masses greater than 10 kg (*Varanus komodoensis*: 72 kg; *Caiman crocodilus*: 22.1 kg), while our crocodylian sample helps to flesh out the upper range of extant reptilian size by adding five additional specimens with masses greater than 10 kg. Measurements were taken either directly on the specimen or digitally using 3-Matic (Supp. File 2). Using OLS, we regressed log10 body mass for this extant dataset (*n* = 266) against log10 combined stylopod circumference. As with volumetric-based estimation, we performed phylogenetic GLS regressions with the stylopodial-scaling approach. However, not all 266 taxa were available in timetree.org, so we included a subset of the 233 available individuals for the phylogenetic GLS analysis. OLS regressions were performed in base R (R Core Team 2024), while GLS regressions used the “gls” function from “nlme” (Pinheiro and Bates 2000).

### Determining accuracy and precision of estimates

To compare accuracy between volumetric-based (*n* specimens = 33, *n* species = 25) and stylopodial-scaling (*n* = 266) estimation among extant taxa, we calculated the PPE for each specimen (Smith 1980; Wimberly 2023) using the “ppe” function from the “MASSTIMATE” package (R Core Team 2024). Density plots of logged PPE were compared for each amniote clade (mammals and reptiles/crocodylians) against the total group to determine whether each method produces accurate within-group body mass estimates. An expectation of within-group accuracy for a universal estimation method would be a within-group PPE distribution that closely approximates the total group PPE distribution. In other words, the regression equation derived from the total group sample is similarly successful at estimating body mass for both mammals and reptiles/crocodylians, whereas a within-group PPE distribution that differs from the total group PPE distribution would suggest clade-specific differences in application of the regression equation.

To compare precision between stylopodial-scaling and MCH estimation, we calculated mean PPE, including 95% confidence intervals, across each group. Because the sample size for stylopodial-scaling was considerably larger than MCH estimation, we randomly sub-sampled 1000 iterations of the stylopodial-scaling analysis to the same sample size as the total volumetric sample (*n* = 33) to determine any influence of sample size.

### Estimating body mass in extinct tetrapods

Our extinct dataset included directly calculating MCH volume estimates for all but three of the 12 fossil tetrapods listed above, and incorporating the published values of *Tapinocaninus* (Romano and Rubidge 2019), *Lisowicia* (Romano and Manucci 2019), and *Bradysaurus* (Brandt et al. 2023). For the nine fossil taxa where we computed volumes directly, we used the same splayed T-pose described for the crocodylians (Fig. 1) and followed the same procedure for generating MCH volumes as the extant sample. As *Edaphosaurus* and *Dimetrodon* both have a dorsal sail, we included this feature as an additional body segment in our analysis. Further, we measured humerus and femur minimum circumference for these nine specimens and used reported values in the literature for the remaining three taxa (*Tapinocaninus, Lisowicia*, and *Bradysaurus*). The extant regression equations for each estimation method were then used to predict the body masses of the 12 fossil taxa, using MCH-computed volumes and combined humerus and femur circumferences, respectively (Supp. File 2). Body mass estimates were then compared between methods.

### Stylopod “Robustness”

In addition to measuring stylopod circumference, we collected humerus and femur length measurements and investigated stylopod “robustness” across our sample, measured as log10 combined stylopodial circumference regressed against log10 combined stylopodial length (*n* = 266 for extant taxa, and *n* = 9 for fossil taxa; the literature did not report stylopod lengths for *Tapinocaninus, Lisowicia*, or *Bradysaurus*) using OLS. To tease apart differences between the forelimb and hindlimb, we performed additional OLS regressions, as above, for solely the humerus and femur, respectively. To examine how robustness scales with body size, we also regressed robustness (circumference/length) against MCH-predicted body mass for combined stylopodial measurements as well as the humerus and femur individually. We used MCH-predicted body mass so that this analysis could be performed on and compared against our fossil sample in addition to the extant taxa.

### Center of mass

Taking advantage of our MCH models, we also quantified the position of the CoM for 12 crocodylians (this study), 6 mammals (Coatham et al. 2021b; excluding *Hemicentetes*, which has no tail), and 9 fossil tetrapods (excluding *Tapinocaninus, Lisowicia*, and *Bradysaurus*) in two ways: (1) the percentage cranial along a transect from the acetabulum to the glenoid and (2) the ratio of the distance cranial to the acetabulum relative to the length of the femur, a metric previously used to inform posture (see Supp. File 1 for a full description of how measurements were performed) (Otero et al. 2019). Specimens were posed in the same way as described for the MCH mass estimations; similarly, because the position of the CoM is calculated relative to the girdles, the degree of limb splay is expected to have negligible impact on the cranial-caudal position of the CoM. To explore the influence of body proportions on cranio-caudal CoM, we regressed each CoM positional proxy against the log10 ratio of skull-to-tail volume (log-transformed to reduce skew).

While previous work has emphasized producing realistic volumetric models for estimating CoM by including, for example, expanded flesh, non-uniform segment densities and airways in the neck and trunk (Bates et al. 2015; Otero et al. 2019), we are more interested here in the comparative value of simplistic models as it relates to the influence of gross body proportions on CoM position. However, to determine whether using MCH models has an impact on broader comparative interpretations, we repeated these analyses on the same specimen sample by “inflating” individual body segments using mammalian-specific expansion factors (MEFs) and sauropsid-specific expansion factors (SEFs) from Coatham et al. (2021b) and Macaulay et al. (2023), respectively. To ensure equivalent implementation, expansion factors were determined by using the raw convex hull:skin values for each body segment and averaged across all mammals (MEFs) or all non-avian sauropsids (SEFs). Mammalian convex hull models were expanded using the MEFs, while crocodylian convex hull models were expanded using SEFs. The Permo-Triassic fossils were expanded using both the MEFs and SEFs to see if these different methods affected interpretations. To further investigate the impact of our choice to model uniform segment density across the body, we repeated our CoM analyses on the unexpanded MCH models with the trunk segment scaled to 90%, 80%, and 70% of the convex hull estimated volume, which is in accordance with experimental measurements of segment density variation (Buchner et al. 1997).

## Results

### Accuracy and precision of body mass estimation methods

**Fig. 2:**
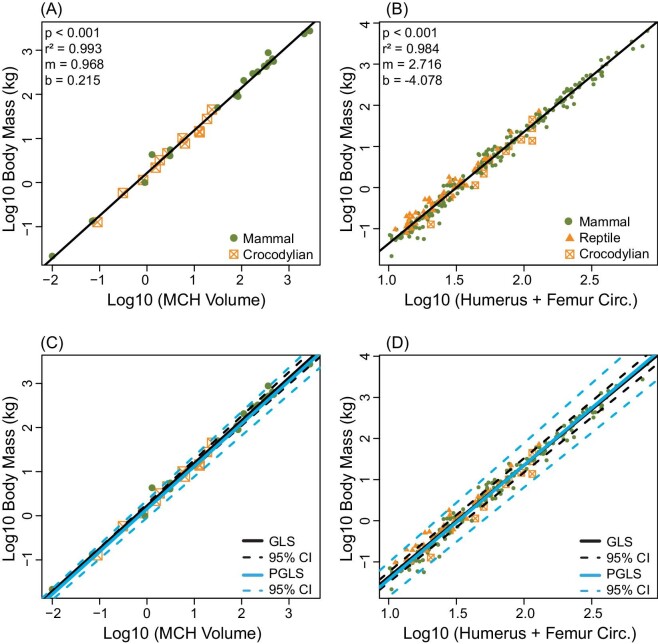
Ordinary least squares regression results for extant taxa showing (A) volumetric-based MCH and (B) stylopodial-scaling body mass estimation methods. Phylogenetic GLS regression (blue) compared to GLS regression (black) for the (C) volumetric-based MCH and (D) stylopodial-scaling body mass estimation methods. Dashed lines in (C and D) represent 95% confidence intervals for each respective model.

Log10 body mass regressed against log10 MCH body mass for our 33 extant specimens exhibited a strong correlation (Fig. 2A; *P* < 0.001; *r*^2^ = 0.993) and was a better model fit than forcing the unlogged slope through the origin, supported by a large reduction in skew apparent from quantile–quantile plots (Supp. Fig. S1). The predictive scaling equation for our volumetric-based model is as follows:


\begin{eqnarray*}
&&{{\log }_{10}}\left( {{\mathrm{Body}}\,{\mathrm{Mass}}} \right) = 0.968\\
&&\qquad *\ {{\log }_{10}}\left( {{\mathrm{MCH}}\,{\mathrm{Volume}}*{\mathrm{Density}}} \right) + 0.215
\end{eqnarray*}

where Density is assumed to be 1000 kg/m^3^. Our updated stylopodial-scaling equation for 266 extant taxa is as follows (Fig. 2B):


\begin{eqnarray*}
&&{{\log }_{10}}\left( {{\mathrm{Body}}\,{\mathrm{Mass}}} \right) = 2.716\\
&&\quad *\ {{\log }_{10}}\left( {{\mathrm{Humerus}} + {\mathrm{Femur}}\,{\mathrm{Circumference}}} \right)-4.078
\end{eqnarray*}

For both MCH and stylopodial-scaling estimation, accounting for shared phylogenetic history had no significant impact on the predictive scaling equation (Fig. 2C and D). For this reason, we deduce that phylogeny has a minimal impact on the broader trends observed here and only report the results of the non-phylogenetic regressions moving forward.

**Fig. 3:**
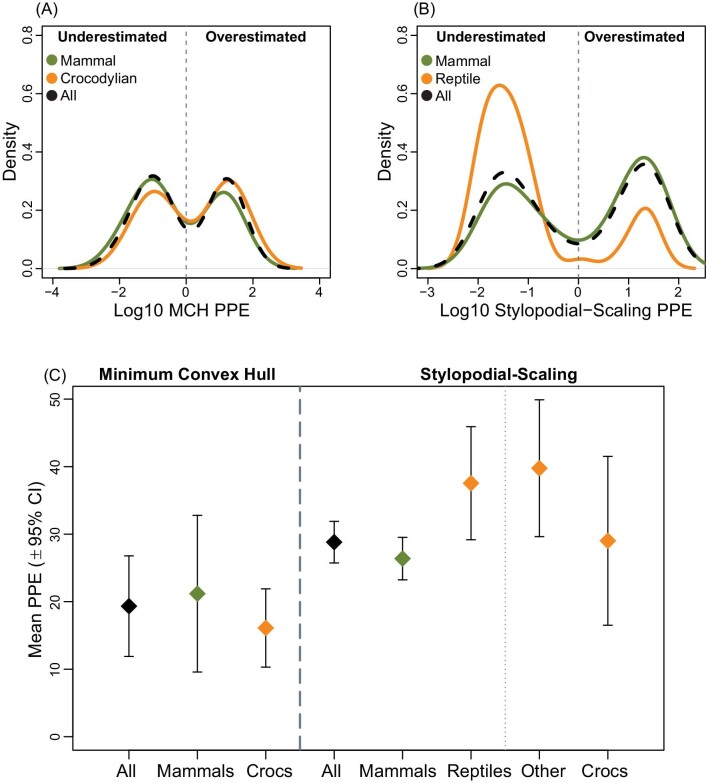
Density plots of logged PPE for (A) volumetric-based MCH and (B) stylopodial-scaling body mass estimation methods. Values left of center indicate individuals where predicted body mass was underestimated compared to specimen body mass, and values right of center indicate individuals where predicted body mass was overestimated compared to specimen body mass. For (A) and (B), the vertical thin dashed black line represents PPE exactly equal to 0. (C) Mean PPE for (left) MCH and (right) stylopodial-scaling estimates of body mass with 95% confidence intervals. The right-most two points for stylopodial-scaling estimation (“Other” and “Crocs”) represent non-crocodylian reptiles (“Other”) and crocodylians (“Crocs”), respectively.

For MCH estimation, both mammals and crocodylians displayed similar distributions around the mean in the PPE density plots, suggesting the model is equally accurate for each group as it is for the total sample (Fig. 3A). For stylopodial-scaling estimation, the body mass of reptiles was more often underestimated, while the body mass of mammals followed the total sample trend, suggesting that the model derived primarily from mammals performs differently within amniote clades (Fig. 3B). These results taken together indicate the accuracy of MCH body mass estimation is more robust to within-clade and between-clade differences.

Regarding precision, MCH mean PPE (19.3 ± 7.4%) was considerably more precise than the stylopodial-scaling mean PPE (28.8 ± 3.1%) for the total sample (Fig. 3C), resulting in an improved precision of 9.5% compared to the stylopod-scaling body mass estimates. This pattern was true for mammals (Fig. 3C; MCH mean PPE = 21.2 ± 11.6%; stylopodial-scaling mean PPE = 26.4 ± 3.1%; improved precision of 5.2%) as well as for crocodylians (Fig. 3C; MCH mean PPE = 16.1 ± 5.8%; stylopodial-scaling mean PPE = 29.0 ± 12.5%; improved precision of 12.9%). The larger confidence intervals using the MCH volumes reflects the smaller sample size; when stylopod circumference dataset was iteratively subsampled (1000 times) to the same sample size as the MCH dataset, the confidence intervals were similar between the two methods, despite consistent differences in mean PPE (Supp. Fig. S2).

### Estimated body mass of Permo-Triassic tetrapods

**Fig. 4:**
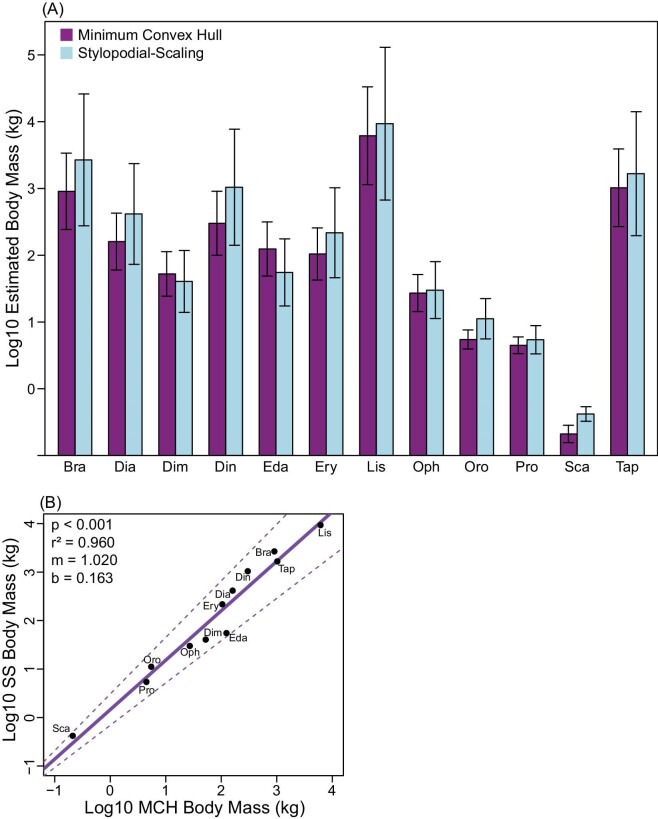
(A) Log10 MCH (purple) and stylopodial-scaling (cyan) estimated body mass for all fossil taxa ± mean PPE. Body masses were estimated from the extant scaling equations; (B) Regression of log10 stylopodial-scaling estimated body mass against log10 MCH-estimated body mass with 95% confidence intervals. Abbreviations: Bra, *Bradysaurus*; Dia, *Diadectes*; Dim, *Dimetrodon*; Din, *Dinodontosaurus*; Eda, *Edaphosaurus*; Ery, *Eryops*; Lis, *Lisowicia*; Oph, *Ophiacodon*; Oro, *Orobates*; Pro, *Procynosuchus*; Sca, *Scaloposaurus*; Tap, *Tapinocaninus*.

With two exceptions (*Dimetrodon, Edaphosaurus*), all fossil taxa sampled had larger estimated body masses using the stylopodial-scaling method compared to the MCH approach (Fig. 4; Table 1; Supp. File 2). When stylopodial-scaling estimated a greater body mass than MCH, this value ranged from 1.1 (*Ophiacodon*: 30 kg/27 kg) to 3.5 (*Dinodontosaurus*: 1042 kg/301 kg) times larger (Fig. 4A). Regressing stylopodial-estimated body mass against MCH-estimated body mass results in a positive slope (Fig. 4B; 1.020 with intercept of 0.163), revealing that the discrepancy between these two estimation methods increases slightly with size.

**Table 1:** Body mass estimates of fossil taxa using the scaling equations from each approach (MCH = Minimum Convex Hull; SS = Stylopodial-Scaling). The table is presented in descending order by the last column, which represents the ratio of stylopodial-estimated body mass to volumetric-estimated body mass (or the discrepancy between the two methods).

**Species**	**Log_10_ MCH Mass**	**MCH Mass (kg)**	**Log_10_ SS Mass**	**SS Mass (kg)**	**Ratio of SS/MCH**
*Dinodontosaurus*	2.5	301	3.0	1042	3.5
*Bradysaurus*	3.0	906	3.4	2673	3.0
*Diadectes*	2.2	160	2.6	415	2.6
*Eryops*	2.0	104	2.3	217	2.1
*Orobates*	0.7	5.5	1.0	11	2.0
*Scaloposaurus*	−0.7	0.2	−0.4	0.4	2.0
*Tapinocaninus*	3.0	1023	3.2	1663	1.6
*Lisowicia*	3.8	6152	4.0	9332	1.5
*Procynosuchus*	0.7	4.5	0.7	5.4	1.2
*Ophiacodon*	1.4	27	1.5	30	1.1
*Dimetrodon*	1.7	52	1.6	41	0.8
*Edaphosaurus*	2.1	124	1.7	55	0.4

### Differences in stylopod robustness

**Fig. 5:**
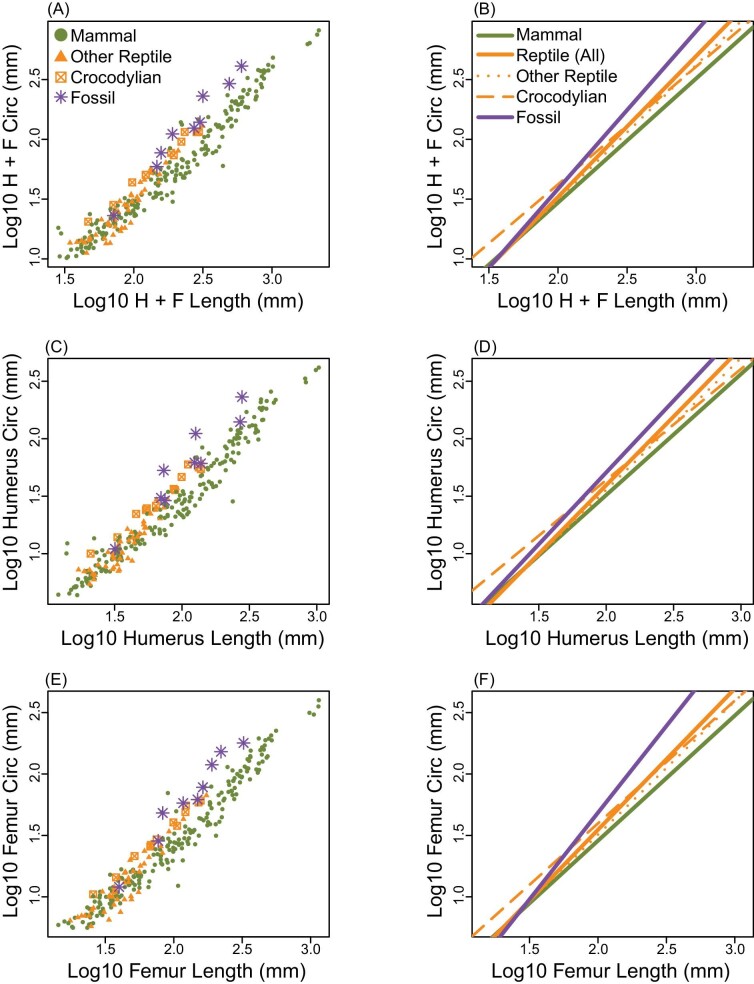
(A, C, E) Log10 stylopod circumference regressed against log10 stylopod length. (B, D, F) Summary regression lines from (A, C, E). (A and B) Combined humerus and femur dimensions; (C and D) humerus only; (E and F) femur only. “Other Reptile” indicates non-crocodylian reptiles.

Stylopod robustness may explain differences between the two mass estimation approaches. The combined humerus and femur dimensions of reptiles in our dataset scale with positive allometry (Fig. 5A and B; Table 2; slope = 1.179 ± 0.102), meaning that reptiles with longer stylopodia have relatively more robust stylopodia. Mammal stylopodia also scale positively (slope = 1.038 ± 0.031), but the slope falls below the 95% confidence intervals of the reptilian slope, indicating that for a given length mammal bones are not as robust as reptiles (Fig. 5A and B; Table 2). Crocodylians scale with isometry instead of positive allometry (slope = 0.985 ± 0.089) and instead have a positively shifted intercept (−0.347 ± 0.194) compared to the intercept of other reptiles (−0.759 ± 0.252), indicating that their stylopodia are simply more robust at all lengths. For combined stylopod dimensions, Permo-Triassic tetrapods scale with positive allometry and have the steepest slope (1.319 ± 0.214).

**Table 2:** Stylopod robustness OLS regression statistics (log10 circumference against log10 length) for mammals, reptiles, and fossils. Significance denoted with (*).

	***P***	***r* ^2^**	**Slope**	**Slope** **95% CI**	**Intercept**	**Intercept** **95% CI**	**Allometry**
Humerus and Femur							
Mammals	0.001*	0.955	1.038	1.007 to 1.069	−0.605	−0.676 to −0.534	Positive
Reptiles (All)	0.001*	0.905	1.179	1.077 to 1.281	−0.840	−1.043 to −0.637	Positive
*Reptiles (Non-Crocs)*	0.001*	0.873	1.127	0.996 to 1.258	−0.759	−1.102 to −0.507	Isometry
*Crocodylians*	0.001*	0.984	0.985	0.896 to 1.074	−0.347	−0.541 to −0.153	Isometry
Fossils	0.001*	0.968	1.319	1.106 to 1.533	−1.053	−1.563 to −0.542	Positive
Humerus							
Mammals	0.001*	0.950	1.053	1.020 to 1.086	−0.596	−0.661 to −0.531	Positive
Reptiles (All)	0.001*	0.900	1.194	1.088 to 1.300	−0.792	−0.969 to −0.615	Positive
*Reptiles (Non-Crocs)*	0.001*	0.881	1.147	1.020 to 1.275	−0.739	−0.945 to −0.534	Positive
*Crocodylians*	0.001*	0.974	0.967	0.856 to 1.079	−0.294	−0.500 to −0.088	Isometry
Fossils	0.001*	0.907	1.270	0.907 to 1.633	−0.821	−1.566 to −0.076	Isometry
Femur							
Mammals	0.001*	0.954	1.019	0.989 to 1.050	−0.581	−0.644 to −0.518	Isometry
Reptiles (All)	0.001*	0.901	1.149	1.048 to 1.251	−0.752	−0.926 to −0.579	Positive
*Reptiles (Non-Crocs)*	0.001*	0.856	1.087	0.952 to 1.223	−0.668	−0.891 to −0.445	Isometry
*Crocodylians*	0.001*	0.990	1.000	0.928 to 1.072	−0.402	−0.541 to −0.264	Isometry
Fossils	0.001*	0.957	1.311	1.062 to 1.560	−0.969	−1.499 to −0.440	Positive

In both mammals and reptiles, the humerus scales at slightly steeper rates (Fig. 5C and D; Table 2; reptile slope = 1.194; mammal slope = 1.053) than the femur (Fig. 5E and F; Table 2; reptile slope = 1.149; mammal slope = 1.019), indicating that femur robustness is more geometrically similar across a wider length range than humerus robustness. Within crocodylians, the humerus and femur scale with only marginally different slopes (0.967 ± 0.112 and 1.000 ± 0.072, respectively), but the humerus does have a positively shifted intercept compared to the femur (−0.294 ± 0.206 and −0.402 ± 0.138, respectively), indicating that the humerus is more robust than the femur across all lengths. The Permo-Triassic tetrapods most closely resemble crocodylians in that the humerus and femur slopes are only marginally different (1.270 ± 0.363 and 1.311 ± 0.249, respectively), but the humerus intercept is shifted positively compared to the femur (−0.821 ± 0.745 and −0.969 ± 0.529, respectively). It is worth noting the wide confidence intervals for the fossil sample, which likely reflects that there are diverse groups represented here (e.g., stem amphibians, stem amniotes, and stem mammals).

**Fig. 6:**
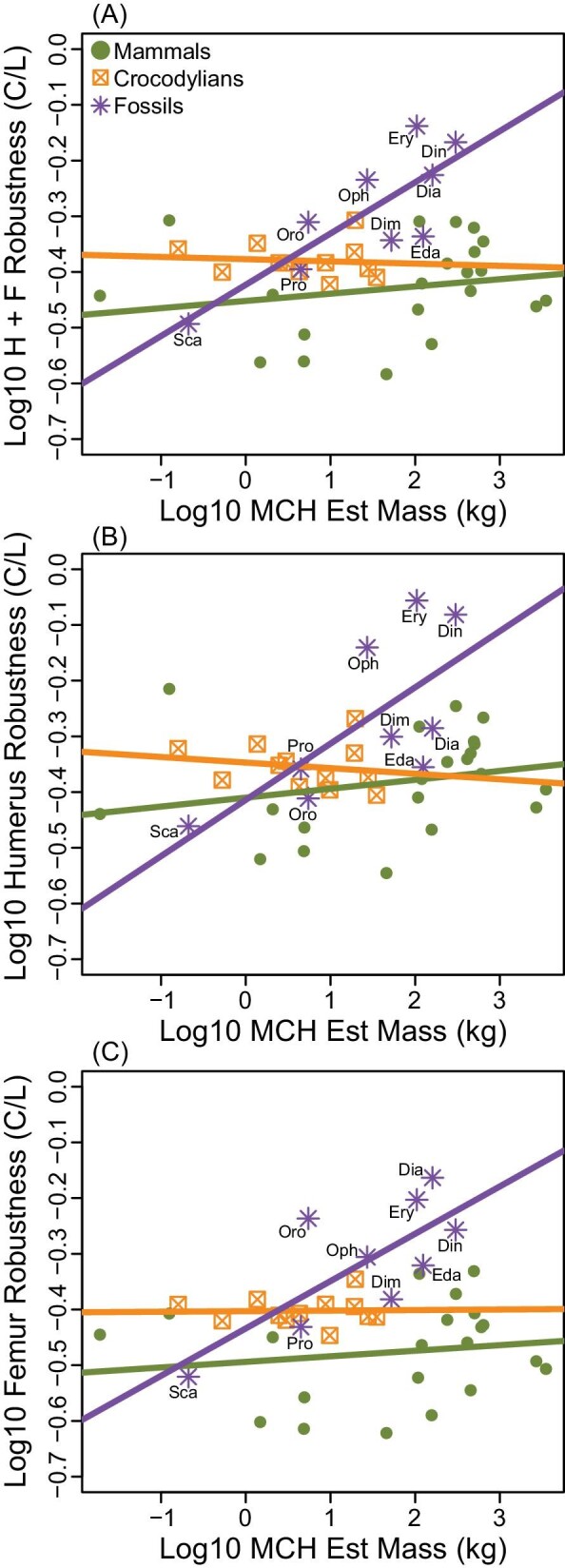
Log10 stylopod “robustness” (circumference/length) against log10 MCH-predicted body mass for (A) combined humerus and femur, (B) humerus, and (C) femur. Abbreviations: Dia, *Diadectes*; Dim, *Dimetrodon*; Din, *Dinodontosaurus*; Eda, *Edaphosaurus*; Ery, *Eryops*; Oph, *Ophiacodon*; Oro, *Orobates*; Pro, *Procynosuchus*; Sca, *Scaloposaurus*.

Regressing log10 stylopod robustness against log10 MCH-predicted body mass (Fig. 6A; Table 3) reveals that mammalian stylopod robustness (slope = −0.013 ± 0.029) and crocodylian stylopod robustness (slope = −0.004 ± 0.030) scale with isometry. Permo-Triassic tetrapod stylopodia scale with positive allometry (slope = 0.092 ± 0.058). These group differences are similar for the humerus (Fig. 6B) and the femur (Fig. 6C). For Permo-Triassic tetrapods, the scaling of humerus robustness regressed against MCH-predicted body mass (slope = 0.101 ± 0.093) has a marginally steeper slope than that of the femur (slope = 0.085 ± 0.067). The intercept for mammals and crocodylians is more variable than the slope, with the humerus shifted positively (−0.410 ± 0.069 and −0.347 ± 0.037, respectively) compared to the femur (−0.494 ± 0.067 and −0.403 ± 0.024, respectively). For Permo-Triassic tetrapods, the intercept is more similar between the humerus (−0.414 ± 0.159) and the femur (−0.434 ± 0.114).

**Table 3:** Log10 stylopod robustness (circumference/length) regressed against log10 MCH-predicted body mass for mammals, crocodylians, and Permo-Triassic fossils using OLS. Significant departure from isometry (expected slope = 0) denoted with (*).

	***P***	***r* ^2^**	**Slope**	**Slope** **95% CI**	**Intercept**	**Intercept** **95% CI**	**Allometry**
Humerus and Femur							
Mammals	0.366	0.043	0.013	−0.016 to 0.042	−0.452	−0.517 to −0.387	Isometry
Crocodylians	0.775	0.009	−0.004	−0.034 to 0.026	−0.377	−0.406 to −0.348	Isometry
Fossils	0.007*	0.667	0.092	0.034 to 0.150	−0.423	−0.522 to −0.325	Positive
Humerus							
Mammals	0.278	0.062	0.016	−0.014 to 0.047	−0.410	−0.479 to −0.341	Isometry
Crocodylians	0.575	0.033	−0.010	−0.049 to 0.029	−0.347	−0.385 to −0.310	Isometry
Fossils	0.038*	0.482	0.101	0.007 to 0.194	−0.414	−0.573 to −0.255	Positive
Femur							
Mammals	0.502	0.024	0.010	−0.020 to 0.040	−0.494	−0.561 to −0.427	Isometry
Crocodylians	0.919	0.001	0.001	−0.023 to 0.025	−0.403	−0.427 to −0.380	Isometry
Fossils	0.020*	0.560	0.085	0.018 to 0.153	−0.434	−0.548 to −0.319	Positive

### CoM position

**Fig. 7:**
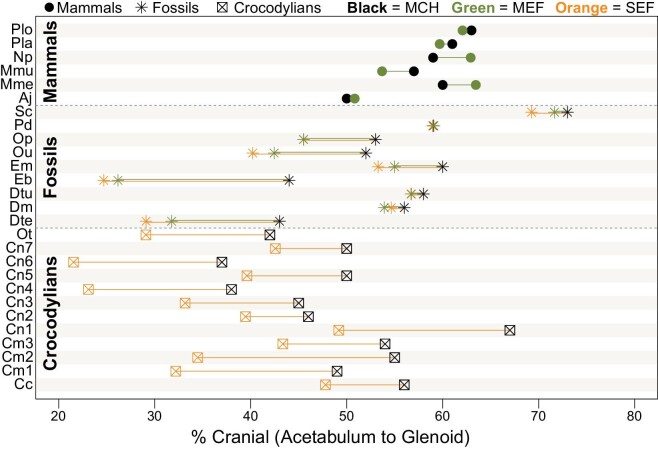
Position of the CoM for all convex hulled specimens in this study. CoM estimated from unexpanded MCH models are indicated in black for each specimen. For mammals, CoM was additionally estimated using MEFs for each body segment. For crocodylians, CoM was additionally estimated using SEFs for each body segment. For Permo-Triassic fossils, CoM was additionally estimated using both MEFs and SEFs for each body segment. MEFs were derived Coatham et al. (2021b) and SEFs from Macaulay et al. (2023). For specimen abbreviations, see Supp. File 2.

**Fig. 8:**
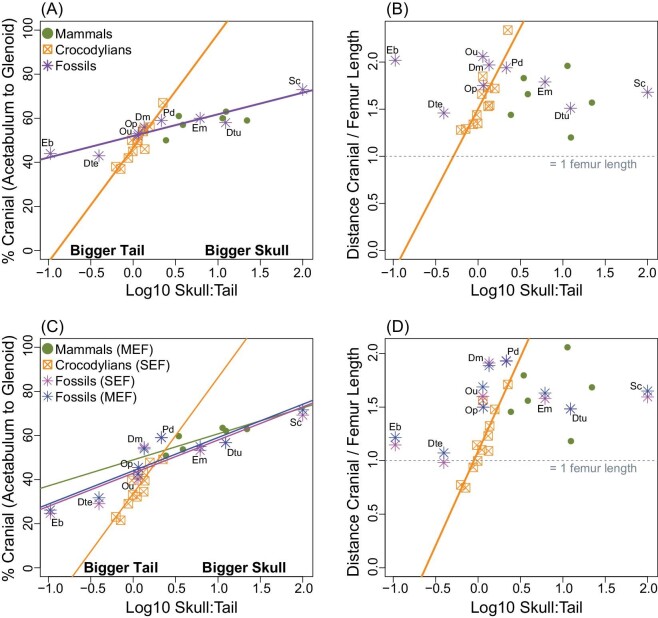
Position of the CoM for mammals (green), crocodylians (orange), and fossil tetrapods (purple) against log10 skull-to-tail volume ratio for (A, C) CoM position measured as the % cranial along a transect from the acetabulum to the glenoid and (B, D) CoM position measured as the ratio of the linear distance cranial to the acetabulum divided by femur length. CoM was calculated directly using unexpanded MCH models (A, B) and by calculating heterogeneous body segment expansion factors (C, D). MEF, mammalian-specific expansion factor; SEF, sauropsid-specific expansion factor. For C and D, CoM of fossils was calculated using both MEFs (pink) and SEFs (violet). Regression lines are plotted only for models where *P* < 0.05. For specimen abbreviations, see Supp. File 2.

The position of the CoM (Fig. 7; Supp. File 2) was dependent on the relative size of the skull and tail in some instances but not others (Fig. 8; Table 4). Percent cranial along a transect from acetabulum to glenoid increased significantly with skull:tail volume for crocodylians (*P* < 0.001, *r*^2^ = 0.871; slope = 51.80 ± 14.05) and extinct taxa (*P* < 0.001, *r*^2^ = 0.887; slope = 9.81 ± 3.13) but not for mammals (*P* = 0.208) using the unexpanded MCH models (Fig. 8A). For crocodylians modeled with the SEFs (Fig. 8C), the intercept decreased in response to a caudally shifted CoM (33.66 ± 2.94 compared to 46.49 ± 2.15), but the slope remained largely the same (52.58 ± 19.22 compared to 51.80 ± 14.05). For the Permo-Triassic fossil sample, irrespective of whether SEFs or MEFs were used to inflate each body segment, the change in relationship was the same: a slightly decreased intercept and a slightly increased slope (Fig. 8C; Table 4). The reason for this change is that taxa with smaller relative skull:tail volumes (i.e., larger tails than skulls) had larger caudal shifts in CoM regardless of which expansion factor was used (e.g., see Eb = *Edaphosaurus* and Dte = *Diadectes* in Figs. 7 and 8C). For mammals, the degree of change when using MEFs to inflate the models was minimal (Fig. 7) but enough to become a statistically significant positive relationship with respect to skull:tail volume (*P* < 0.032, *r*^2^ = 0.721) (Fig. 8C; Table 4). Distance of the CoM cranial from the acetabulum scaled to femur length was instead largely unaffected by using the heterogeneously expanded body segment models, except that three of the crocodylians now have CoM positions that are estimated to be less than one femur length cranial to the acetabulum (Fig. 8D compared to Fig. 8B). The intercepts for the crocodylians and fossils decreased in response to the caudally shifted CoM, but the slopes and overall regressions found no observable differences between the unexpanded and expanded models for any group (Table 4). Finally, deflating the trunk volume to 90, 80, and 70% had a negligible impact on any of these patterns (Supp. File 2).

**Table 4:** Center of mass (CoM) OLS regression summary statistics for Minimum Convex Hull (MCH) and expanded (Exp.) models using the mammal-specific expansion factor (MEF) and sauropsid-specific expansion factor (SEF). %C(AG) = percentage cranial the CoM is from the acetabulum to the glenoid. DCA/FL = the distance cranial from acetabulum relative to femur length. Both metrics were regressed against log10 skull:tail volume ratio. Significance denoted with (*).

	***P***	***r* ^2^**	**Slope**	**Slope 95% CI**	**Intercept**	**Intercept 95% CI**
%C(AG) MCH						
Mammals	0.208	0.360	7.18	−6.10 to 20.44	52.35	40.36 to 64.34
Crocodylians	0.000*	0.871	51.80	37.75 to 65.86	46.49	44.34 to 48.64
Fossils	0.000*	0.887	9.81	6.68 to 12.94	51.98	49.20 to 54.75
DCA/FL MCH						
Mammals	0.851	0.010	−0.07	−1.06 to 0.92	1.67	0.78 to 2.56
Crocodylians	0.000*	0.747	1.73	1.02 to 2.44	1.50	1.39 to 1.61
Fossils	0.332	0.134	−0.09	−0.30 to 0.12	1.83	1.64 to 2.02
%C(AG) Exp.						
Mammals (MEF)	0.032*	0.721	11.80	1.61 to 21.99	48.93	39.72 to 58.14
Crocs (SEF)	0.000*	0.788	52.58	33.36 to 71.80	33.66	30.71 to 36.60
Fossils (SEF)	0.001*	0.806	15.04	8.44 to 21.65	42.88	37.03 to 48.74
Fossils (MEF)	0.000*	0.851	15.10	9.46 to 20.74	43.97	38.98 to 48.96
DCA/FL Exp.						
Mammals (MEF)	0.865	0.008	0.07	−1.02 to 1.16	1.56	0.58 to 2.55
Crocs (SEF)	0.000*	0.776	1.76	1.09 to 2.42	1.09	0.98 to 1.19
Fossils (SEF)	0.254	0.181	0.15	−0.14 to 0.44	1.47	1.22 to 1.73
Fossils (MEF)	0.245	0.187	0.14	−0.12 to 0.41	1.51	1.28 to 1.75

## Discussion

Here, we find that MCH body mass estimation (Fig. 2A) is both more accurate and more precise than universal stylopodial-scaling body mass estimation (Fig. 2B). In comparison to stylopodial-scaling, MCH is negligibly affected by within-clade and between-clade variability (Fig. 3A and B), and it reduces body mass estimation error (4.4% for mammals and 13.0% for crocodylians), supporting higher precision (Fig. 3C). For the MCH approach, PPE was distributed similarly around the mean for both groups (mammal and crocodylians) indicating high accuracy. For stylopodial-scaling, the PPE density distribution for mammals matched the total sample (high accuracy), reflecting the mammalian bias in the prediction equation, but was highly skewed to the left in reptiles (low accuracy). Therefore, our findings contradict recent suggestions that universal stylopodial-scaling provides a more accurate method of estimating body mass than whole-body volumetric models (Campione and Evans 2012, 2020). An explanation for the improved accuracy and precision of the MCH approach is that limb bone dimensions scale differentially across amniotes. At larger sizes, the relatively more robust humeri and femora (circumference/length) of reptiles result in overestimation of body mass when using the mammalian-weighted prediction equation, while the body mass of smaller reptiles with less robust stylopodia is underestimated (Figs. 3 and 5; Table 2). Instead, MCH body mass estimation is less influenced by individual bone scaling patterns because it incorporates more information about a skeletonized animal than is available from a single or few elements (Brassey 2016).

Animals are known to deal with issues of limb stress (e.g., due to body size or fast running speeds) by adjusting aspects of their musculoskeletal system. Mammals, with parasagittal limb postures, reduce musculoskeletal stresses by extending their limb joints as size increases, and only very large mammals (> ∼300 kg) increase stylopod robustness (Biewener 1989, 1990). In contrast, modern sprawling reptiles, which are smaller than the largest mammals, have not evolved the same degree of limb extension and instead reduce stress by increasing stylopod robustness (Christian and Garland 1996). Among sprawling reptilian clades, multiple additional solutions for mitigating size-related limb bone stress have evolved in different groups (Blob 2000). For instance, varanid lizards have stylopodia that scale with positive allometry (Dick and Clemente 2017) and further reduce stress by increasing duty factor and decreasing femoral rotation with size (Clemente et al. 2011; Cieri et al. 2021). Among our limited sample, crocodylians have a positively shifted stylopod robustness scaling intercept, indicating more robust bones than other clades across all sizes (Fig. 5; Table 2). Previous work suggests that limb bone scaling is variable across crocodylian species (Iijima and Kubo 2019), and these animals perhaps instead, as is the case for the American alligator, favor changes to posture (partially adducted and extended limbs) and mechanical work (shorter, faster strides) as body mass increases (Iijima et al. 2023). All of these clade-specific scaling patterns influence how stylopod dimensions relate to body mass and thus reduce both the accuracy and precision of universal element-scaling equations applied to individual clades.

For paleobiologists seeking to estimate the body masses of extinct tetrapods, the difference between MCH and stylopodial-scaling can be profound, and our findings are consistent with the literature in this regard (Bates et al. 2015; Romano and Manucci 2019; Romano and Rubidge 2019; Hart et al. 2022). Applied to our sample of 12 Permo-Triassic tetrapods, stylopodial-scaling consistently estimates larger body masses than MCH, and the discrepancy increases with size (Fig. 4; Table 1). At its largest difference, stylopodial-scaling estimates a mass 3.5 times the size of MCH-scaling estimates (*Dinodontosaurus*: 1042 kg compared to 301 kg). Because these animals predominantly had sprawling postures and were larger than most extant sprawling animals, they likely had to evolve relatively more robust limb bones, in addition to any potential posture or behavior-related changes, to mitigate size-related stress (Fig. 5; Table 2). Increased scaling of limb bone robustness compared to extant taxa exacerbates the error associated with extant stylopodial-scaling body mass estimates. Overestimation of body mass is problematic because it can alter our interpretations of extinct animal biology (e.g., how fast an animal might run or how high it can lift its body off the ground) (Hutchinson et al. 2007). Interestingly, two species in our sample with atypical morphologies—a sail-like structure on their back (*Edaphosaurus* and *Dimetrodon*)—have more similar agreement between body mass estimation methods, which was unexpected. One potential reason for this result is that these two species represent gracile members to their respective clades (Romer and Price 1940) and thus may have smaller than expected limb bones, reducing stylopodial-scaling estimates. To identify appropriate clade-specific limb bone robustness scaling, a much wider sample should be examined within each extinct group, though that is beyond the scope of the present investigation.

In addition to body mass, volumetric models also permit the calculation of inertial-related parameters such as CoM, which influence animal posture, gait, energetics, and performance metrics such as turning or running speed (Allen et al. 2009). Quantifying CoM for extinct animals further helps us understand how changes in body plans are related to macroevolutionary patterns (Bates et al. 2016). Here, our simplistic MCH models suggest that quadrupeds have a cranially shifted CoM, typically between 40 and 60% of the distance between the acetabulum and glenoid (Fig. 7; Supp. File 2). However, heterogeneously inflating body segments using mammalian-specific (MEFs; Coatham et al. 2021b) and sauropsid-specific expansion factors (SEFs; Macaulay et al. 2023) shifted this range to 20–60% cranial of the acetabulum, a result primarily driven by the large-tailed crocodylians but also select Permo-Triassic tetrapods (e.g., *Edaphosaurus* CoM moved caudally from 44% to 25–26% and *Diadectes* from 43% to 29–32%) (Fig. 7; Supp. File 2). Both the unexpanded and expanded MCH models recovered CoM positions that were at least one femur length cranial to the acetabulum, which is consistent with a quadrupedal as opposed to bipedal posture as proposed by Otero et al. (2019), except for three of the heterogeneously expanded crocodylian specimens (Fig. 8C and D; Supp. File 2). The relative size of the skull compared to the tail has notable influence on the cranio-caudal position of the CoM for crocodylians and Permo-Triassic fossils regardless of using unexpanded or expanded models, though the slopes and intercepts do change marginally, and for mammals the relationship becomes minimally significant when using the expanded models (Fig. 8A and C; Table 4). These results suggest that group differences are retained whether using unexpanded MCH models or clade-specific expansion factors, though the specific relationship within each group is likely more exact when using the heterogeneously expanded body segment models. For the Permo-Triassic fossil sample, which contains great diversity in morphology and body proportions, using the expanded models to estimate CoM position becomes more important, especially in specimens with notable disparity outside the bounds of the modern sample. For example, contrast the small-headed and large-tailed “pelycosaur” *Edaphosaurus*, which showed a large caudal CoM shift in the expanded models (∼18–19%), to the more crownward and largely mammalian body proportions of *Procynosuchus*, which showed less than 1% variation in CoM position across all the models compared here (Fig. 7; Supp. File 2). Finally, during evolution along the synapsid-mammal line, it has been hypothesized that therapsids evolved a cranially shifted CoM in conjunction with an enlarged head and reduced tail (Jones et al. 2019; Bishop and Pierce 2023; Kaiuca et al. 2024). Compared to more basal synapsids in our sample (*Dimetrodon, Ophiacodon, Edaphosaurus*), therapsids (*Dinodontosaurus, Scaloposaurus, Procynosuchus*) have more cranially shifted CoM (Figs. 7 and 8) irrespective of whether the unexpanded or expanded MCH models are analyzed. This result does not hold when normalized against femur length (Fig. 7B and D), suggesting that limb bones and whole-body morphology change independently of one another, which complicates our understanding of the evolution of CoM position.

There are a few study limitations that should be noted. First, with respect to the ontogenetic nature of our crocodylian sample, it is worth adding a brief cautionary note as ontogeny is thought to explain at least a portion of the difference between volumetric-based and stylopodial-scaling estimation (Bates et al. 2015; Brassey et al. 2015) and is also known to affect body proportions during growth (Thompson 1917). Among crocodylians specifically, a recent study on the American alligator found a relationship with size such that larger individuals had a caudally shifted CoM (Iijima et al. 2023), perhaps demonstrating the increasing importance during growth of the large retractor muscles that extend along the tail (Gatesy 1990). That said, our CoM estimates for the crocodylian sample, as well as the amount that the CoM shifted using heterogeneously expanded models, were independent of size (Fig. 7, Supp. File 2). Second, as described in the methods, the mammalian sample from Sellers et al. (2012) does not include known masses, which are instead derived from the literature. Our view is that species-specific scaling equations are sufficiently distinct from clade-wide scaling equations that these likely reflect realistic body mass values, but it would certainly be an improvement to have masses directly measured from the same specimens that are modeled. Finally, for the specimens we modeled, we manually straightened the tail into one segment rather than subdividing it into multiple segments, as is the case for the mammals from Coatham et al. (2021b). While it is possible that this difference in approach has a very small effect on body mass and CoM calculations, these are probably inconsequential across large taxonomic samples. Nonetheless, we did investigate this by subdividing the tail of our *Caiman* specimen into four segments and found, as would be expected, the MCH-estimated body mass decreased (from 1.53 to 1.49 kg) and the CoM shifted cranially (from 56 to 58% AG). While this confirms tail subhulling can impact estimated values, these are very minimal in the context of the total sample and the aims of the current study.

### Conclusion

To conclude, we find that volumetric-based body mass estimation using the MCH approach is both more accurate and more precise than universal stylopodial-scaling estimation. Stylopod dimensions scale differently among reptiles, mammals, and Permo-Triassic taxa, and improperly accounting for a mammalian-weighted validation sample influences mass predictions in a way that likely overestimates the body mass of extant and extinct non-mammals. While we acknowledge the difficulty in aggregating a large sample size for volumetric-based estimation, we also recommend that element-scaling should be used with caution, particularly when reconstructing the body mass of extinct taxa with anatomies outside the bounds of modern comparators where differences in estimation approach have greater consequence. We encourage continued efforts in building more whole-body models of modern and extinct taxa to help further elucidate the scaling relationships between limb bone dimensions and body mass. Understanding this relationship may provide a means to better predict body mass from isolated fossil elements.

## Supplementary Material

obae034_Supplemental_Files

## Data Availability

The data underlying this article are available in the article and in its online supplementary material.
