# Supplementary material for: Volumetric versus Element-scaling Mass Estimation and Its Application to Permo-Triassic Tetrapods
Source: Integr Org Biol. 2024 Sep 13;6(1):obae034. doi: 10.1093/iob/obae034 (PMC11438236; doi:10.1093/iob/obae034)
Supplement: obae034_Supplemental_Files [file obae034_supplemental_files.zip › Supplementary File 1.docx]

# Supplementary File 1

## Center of Mass Calculations

For center of mass calculations, the locations of the acetabulum and glenoid were identified by placing a digital marker or line through each socket in Materialise 3-Matic and determining its three-dimensional coordinate. The coordinate values for center of mass, glenoid position, and acetabulum position were then considered as the vertices of a triangle. Each side length of the triangle was calculated using the equation

√ [(X_1_ - X_2_)^2^ + (Y_1_ - Y_2_)^2^ + (Z_1_ - Z_2_)^2^)]

where each (X, Y, Z) triplet represents one of the vertices. The area of the triangle was calculated using Hero’s formula:

Area = √ [s(s - a)(s - b)(s - c)]

where s is the semi-perimeter of the triangle, and a, b, and c are the side lengths of the

triangle. The height, h, of the triangle was subsequently calculated using the formula

h = 2 x Area/base

where the base of the triangle is defined as the line from the glenoid to the acetabulum.

The height therefore represents a line segment originating from the center of mass location, that bisects the line from the glenoid to the acetabulum. The location of that intersection is used to determine the relative cranial or caudal position of the center of mass along the glenoid-acetabulum axis, by calculating a right triangle with sides represented by the height calculated in the last step (h); the line between the center of mass and the acetabulum position (side AC); and the line from the intersection of the height and the glenoid-acetabulum axis with the location of the acetabulum (segment DC). The height and the side AC are known and can be used to calculate the length of DC using Pythagoras’s theorem; segment BD can then be determined by inference. The ratio of BD:BC expresses the center of mass as a fraction cranial from the acetabulum to the glenoid.

In order to assess the effect of individual body segments on the results, the normalized center of mass was compared to the relative skull and tail mass ratio, defined by

log_10_((skull volume)/(tail volume))

To ensure that the relationship in this linear model is not impacted by phylogenetic similarity of different specimens, the phylogenetic independent contrast method (PIC) was applied to this regression in RStudio using the “ape” library and the “phytools” package (Revell, 2012). A composite tree of 8 species (excluding *O. pabsti*) was constructed by pruning time-scaled master phylogenies of fossil synapsids and tetrapods from Jones et al, 2019 and Dickson et al, 2020, respectively (Dickson et al., 2021; Jones et al., 2019). For *O. pabsti*, age and divergence from *D. tenuitectus* were sourced from Kissel 2010 (Kissel, 2010). The final composite phylogeny was generated using Mesquite v3.61 and the 'ape' package in R (R Core Team, 2020).

# Supplementary Figures


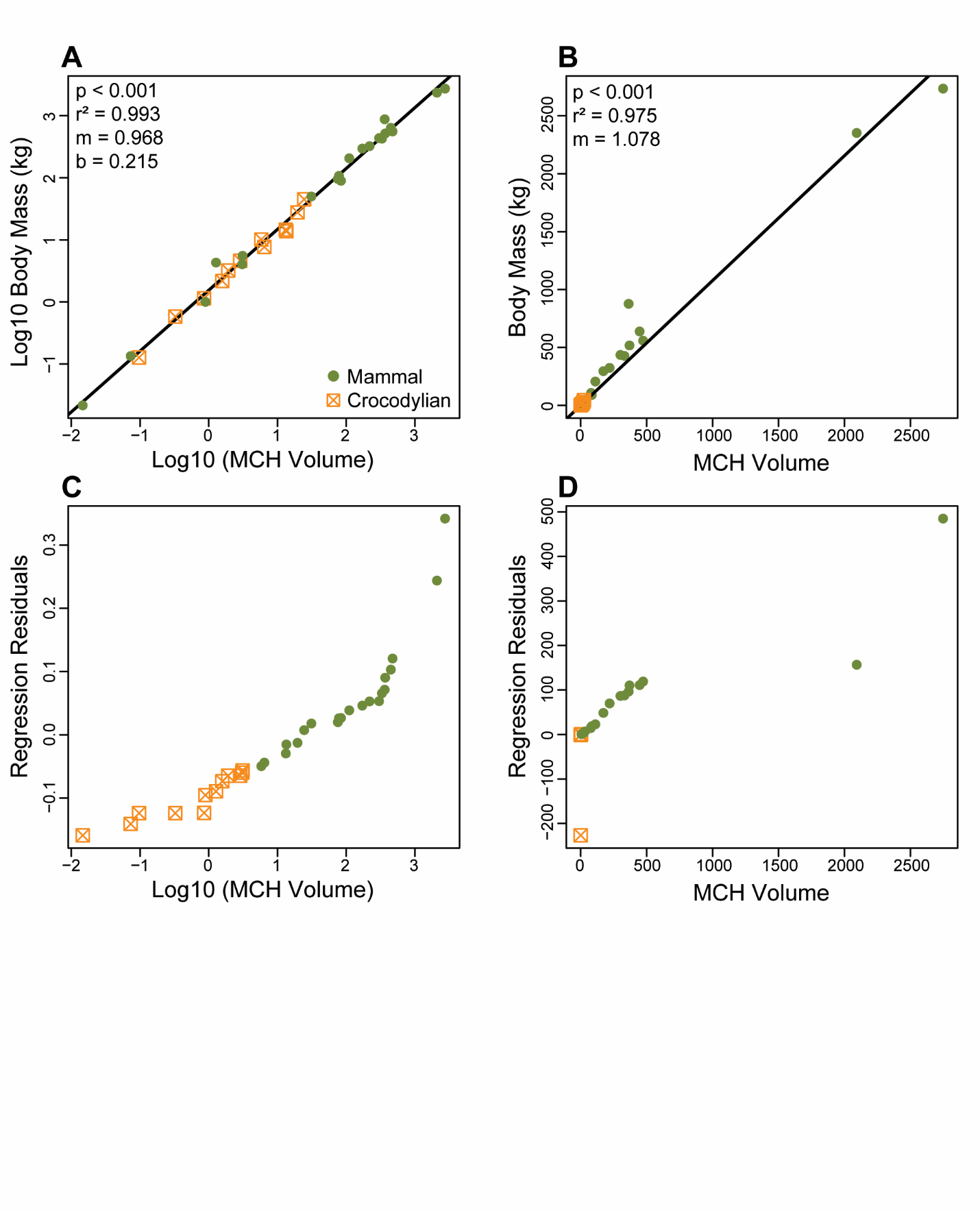


**Supplementary Figure 1**: (A) Regression of log10 body mass against log10 MCH-estimated body mass for all individuals in our sample; (B) Regression of body mass against MCH-estimated body mass with the slope forced through (0,0); (C) Residuals for (A) plotted against logged MCH-estimated body mass; (D) Residuals for (B) plotted against MCH-estimated body mass. Green = mammals; orange = crocodylians.


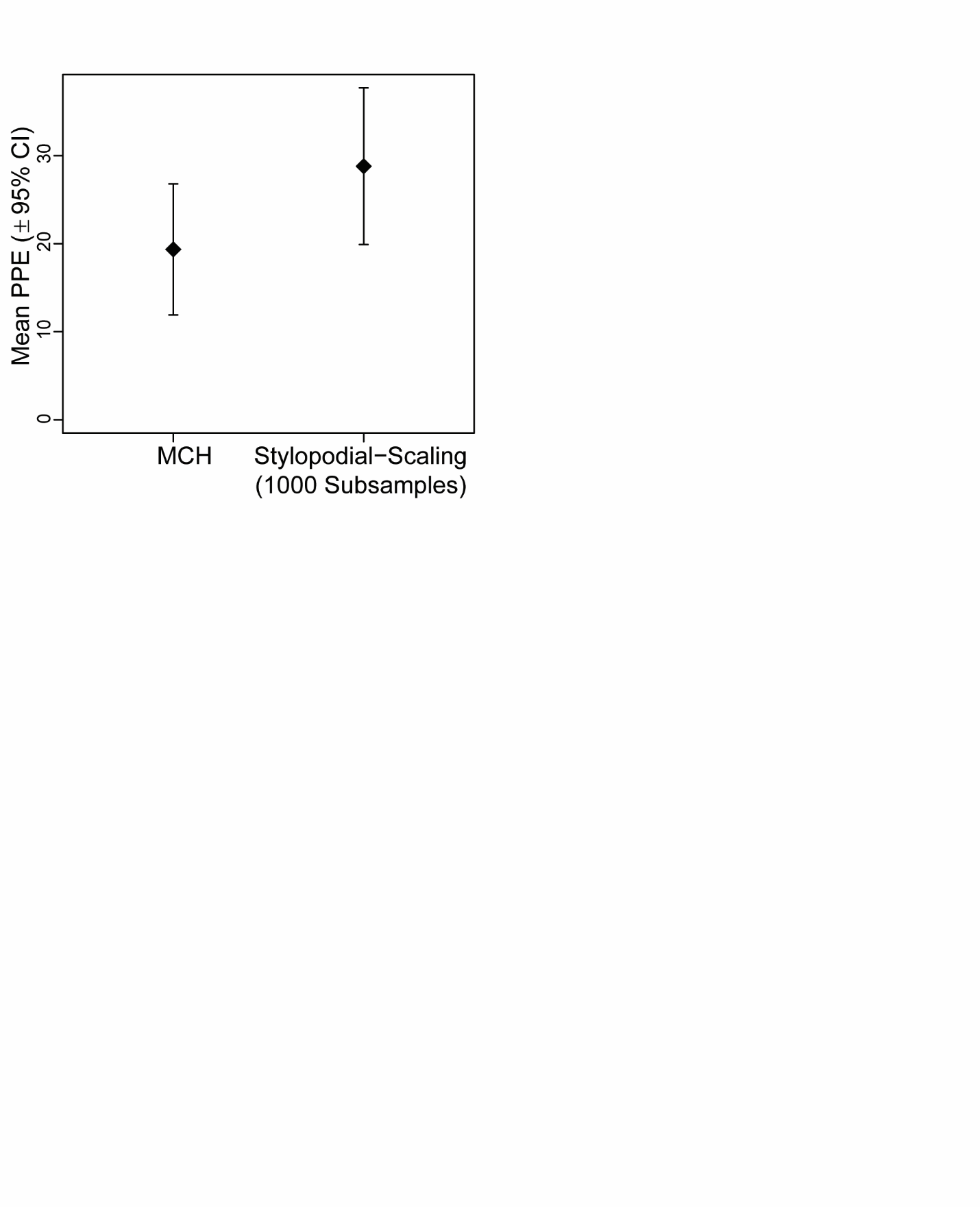


**Supplementary Figure 2**: Mean PPE and confidence intervals for 1000 randomly sub-sampled stylopodial-scaling datasets compared to the original MCH-estimated mean PPE. Confidence intervals show a similar range when sample size is equivalent, yet the error remains consistently high in stylopodial-scaling estimation compared to volumetric-based MCH estimation.


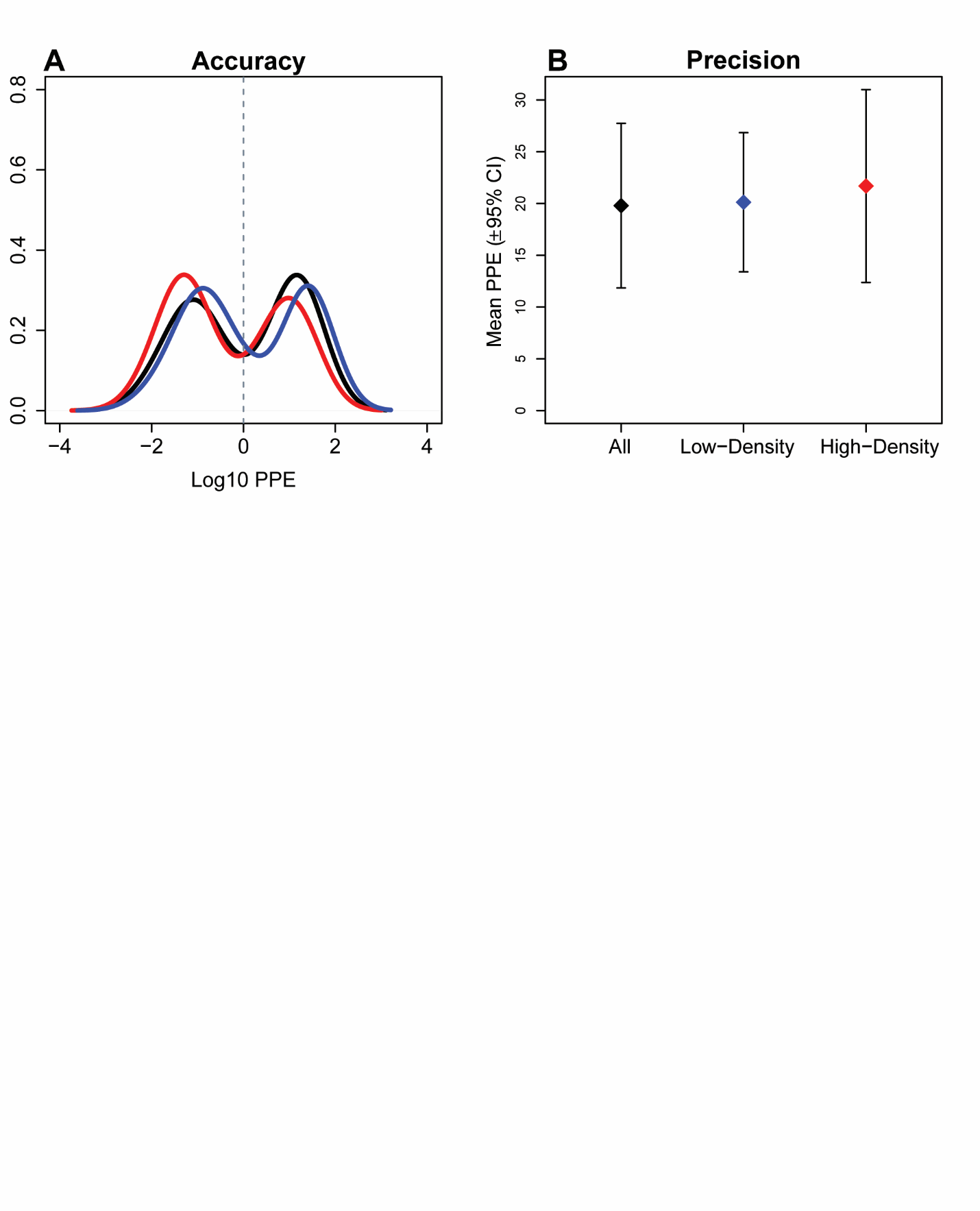


**Supplementary Figure 3**: (A) Accuracy and (B) Precision plots (akin to Figure 3) for (blue) low density estimates (893 kg/m^3^), (red) high density estimates (1,080 kg/m^3^), and (black) a density estimate of water (1,000 kg/m^3^) demonstrating the minimal consequence of varying density within this range.


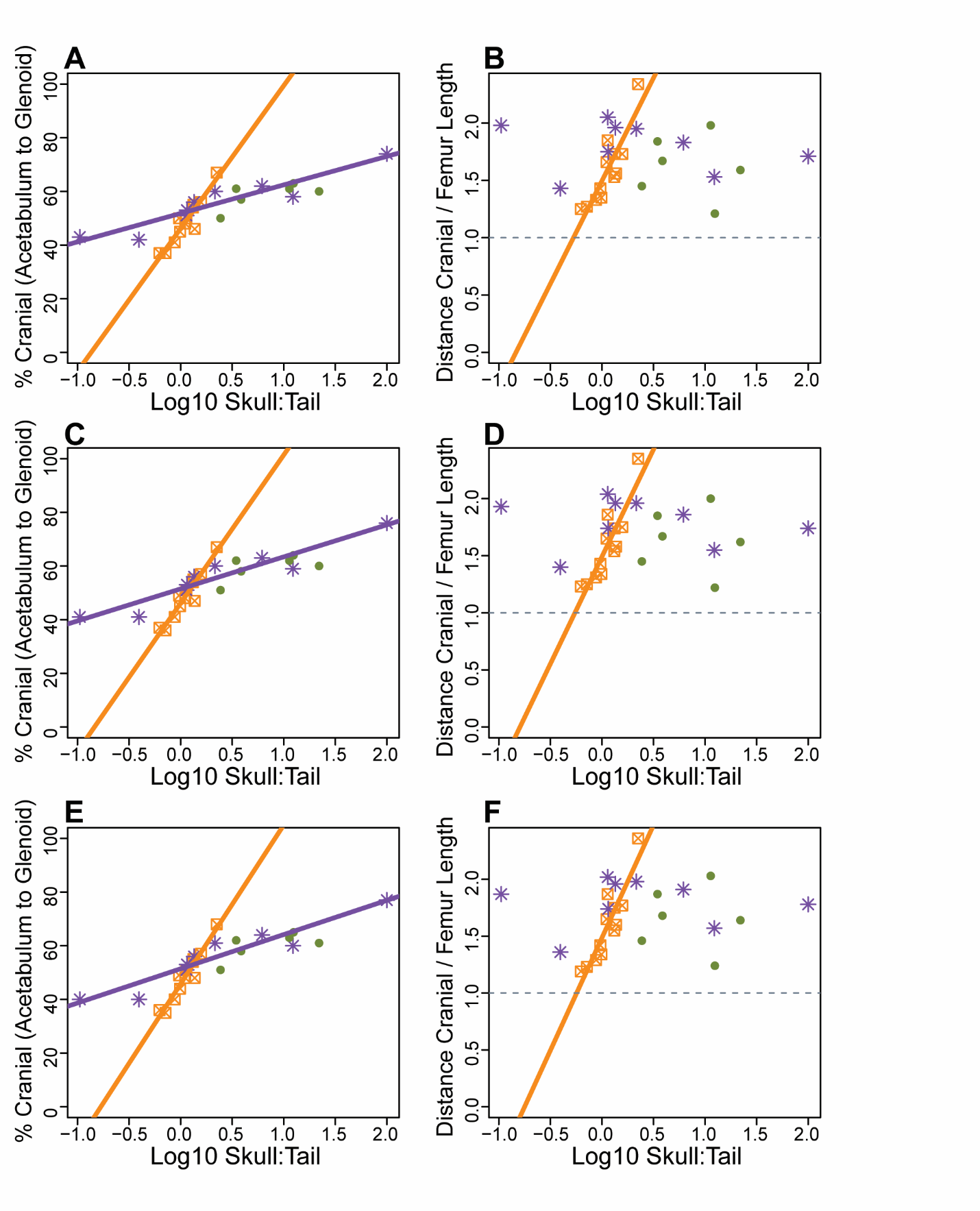


**Supplementary Figure 4**: Position of the center of mass (CoM) for mammals (green), crocodylians (orange) and fossil tetrapods (purple) against log10 skull-to-tail volume ratio for (A, C, E) CoM position measured as the % cranial along a transect from the acetabulum to the glenoid and (B, D, F) CoM position measured as the ratio of the linear distance cranial to the acetabulum divided by femur length. Analyses were repeated with the trunk of each animal adjusted to be 90% (A, B), 80% (C, D), and 70% (E, F) of its convex hull volume. There is negligible change to CoM position with respect to variation in trunk volume (Supp. File 2).

# Supplementary File 2

This file contains the raw data for each specimen in both major foci of this study: body mass estimation and center of mass estimation. The tab “Limb and Volume Data” includes specimen IDs, body mass, limb measurements, whole-body MCH volume, and the estimated mass from both the MCH and stylopodial-scaling approaches. The tab “CoM Data” includes specimen abbreviations, skull and tail segment volumes, and the CoM position estimated from each the unexpanded MCH models, the expanded models, and the trunk-deflated models (to 90%, 80%, and 70% of its original volume).
